# Structural insights into the catalytic mechanism of aldehyde-deformylating oxygenases

**DOI:** 10.1007/s13238-014-0108-2

**Published:** 2014-12-09

**Authors:** Chenjun Jia, Mei Li, Jianjun Li, Jingjing Zhang, Hongmei Zhang, Peng Cao, Xiaowei Pan, Xuefeng Lu, Wenrui Chang

**Affiliations:** 1 National Laboratory of Biomacromolecules, Institute of Biophysics, Chinese Academy of Sciences, Beijing, 100101 China; 2 Key Laboratory of Biofuels, Shandong Provincial Key Laboratory of Energy Genetics, Qingdao Institute of Bioenergy and Bioprocess Technology, Chinese Academy of Sciences, Qingdao, 266101 China; 3 University of Chinese Academy of Sciences, Beijing, 100049 China

**Keywords:** aldehyde-deformylating oxygenase, di-iron center, crystal structure, catalytic mechanism, alk(a/e)ne production

## Abstract

The fatty alk(a/e)ne biosynthesis pathway found in cyanobacteria gained tremendous attention in recent years as a promising alternative approach for biofuel production. Cyanobacterial aldehyde-deformylating oxygenase (cADO), which catalyzes the conversion of C_n_ fatty aldehyde to its corresponding C_n-1_ alk(a/e)ne, is a key enzyme in that pathway. Due to its low activity, alk(a/e)ne production by cADO is an inefficient process. Previous biochemical and structural investigations of cADO have provided some information on its catalytic reaction. However, the details of its catalytic processes remain unclear. Here we report five crystal structures of cADO from the *Synechococcus elongates* strain PCC7942 in both its iron-free and iron-bound forms, representing different states during its catalytic process. Structural comparisons and functional enzyme assays indicate that Glu144, one of the iron-coordinating residues, plays a vital role in the catalytic reaction of cADO. Moreover, the helix where Glu144 resides exhibits two distinct conformations that correlates with the different binding states of the di-iron center in cADO structures. Therefore, our results provide a structural explanation for the highly labile feature of cADO di-iron center, which we proposed to be related to its low enzymatic activity. On the basis of our structural and biochemical data, a possible catalytic process of cADO was proposed, which could aid the design of cADO with improved activity.

## Introduction

Fatty alk(a/e)nes are major components of fuel oil and one of the ideal alternatives for fossil-based biofuels. Recently, Schirmer et al. identified two genes from cyanobacteria that encode an acyl-ACP reductase (AAR) and an aldehyde-deformylating oxygenase (ADO), both of which are responsible for alkane production in cyanobacteria (Li et al., 2012; Schirmer et al., 2010). This pathway has drawn considerable attention, since this route provides a promising approach for photosynthetic production of alka(e)ne biofuels (Krebs et al., 2011; Wang et al., 2013). In this two-step pathway, AAR reduces fatty acyl-ACPs or -CoAs into their corresponding aldehydes, and subsequently the C_n_ fatty aldehydes are converted into their corresponding C_n-1_ alk(a/e)nes by ADO (Schirmer et al., 2010).

Cyanobacterial aldehyde-deformylating oxygenase (cADO) belongs to the superfamily of ferritin-like di-iron proteins (Krebs et al., 2011) and contains a di-iron center (Das et al., 2011). However, metal analysis indicated that less than one fifth of the purified protein in a typical preparation contains iron (Das et al., 2011), assuming two iron atoms per enzyme molecule, the results indicated that the iron atoms may be loosely bound to cADO. The *in vitro* reaction catalyzed by cADO requires both the dioxygen as co-substrate and the presence of a reducing system, which provides four electrons per turnover (Warui et al., 2011) and can either be biological (ferredoxin, ferredoxin reductase, and NADPH; Fd/FR/N) (Schirmer et al., 2010; Warui et al., 2011) or chemical (phenazine methosulfate and NADH; P/N) (Das et al., 2011). It was proposed that the incorporation of O_2_ into the reduced cofactor generates an iron-peroxo species that attacks the substrate aldehyde to form a hemiacetal and followed by the scission of its C_1_–C_2_ bond (Li et al., 2012; Li et al., 2011). The ensuing product of the reaction is a C_n-1_ alkane (Li et al., 2011), and the C_1_-derived co-product was demonstrated to be formate (Warui et al., 2011). Isotope-tracer assay revealed that the oxygen atom in the product formate originates from the co-substrate O_2_ (Li et al., 2012). Recent reports suggested that cADO also catalyzes the incorporation of an oxygen atom from O_2_ into its alkane product for C_9–10_ aldehyde substrates, which yields both C_n-1_ alcohol and aldehyde products, implying a function for cADO in oxygenation, in addition to deformylation (Aukema et al., 2013; Das et al., 2014). An intriguing feature of cADO is its low *in vitro* activity (Das et al., 2011; Eser et al., 2012; Li et al., 2011), with only 3–5 turnovers (Andre et al., 2013; Warui et al., 2011). Andre et al. reported that cADO is reversibly inhibited by the side product H_2_O_2_, and its inhibition can be relieved by adding catalase in enzyme assays (Andre et al., 2013). Subsequently, it was demonstrated that the output of H_2_O_2_ can be reduced by over 30% in the presence of a cognate biological reducing system Fd/FR/N (Zhang et al., 2013). In addition, when cADO was fused with cognate FR and Fd in a specific order, it displayed a 3-fold increase in activity relative to native cADO (Wang et al., 2014).

Crystal structures of ADO from cyanobacterium *Prochlorococcus marinus* (*Pm*) MIT9313 (wild-type and two single-point mutants) were reported (Khara et al., 2013). Structures showed that *Pm*ADO adopted an α-helical folding, with two iron atoms coordinated by histidines and carboxylate ligands (Khara et al., 2013; Schirmer et al., 2010). A long-chain ligand, which was subsequently identified as a mixture of fatty acid molecules (Khara et al., 2013), was observed in the vicinity of the di-iron center in these structures (Khara et al., 2013). This pocket accommodating fatty acid molecules were suggested to be the substrate channel. However, due to the structural difference between fatty acid and the real substrate fatty aldehyde, the interaction between ADO and the substrate remains elusive. In addition, their structural resemblance indicated that they may represent one similar state of the reaction process, thus structures of cADO at different dynamic stages during its reaction cycle will be of great help for understanding its detailed catalytic process.

To gain insights into the reaction catalyzed by cADO, we determined structures of wild type ADO from *Synechococcus elongates* PCC7942 (*Se*) with different treatment (WT0, no treatment; WT1, co-crystallized with iron; WT-HP, soaked in H_2_O_2_), and solved the structures of two *Se*ADO mutants (Y122F and F86YF87Y). Comparison of all our structures revealed that their overall conformations are closely resembling each other, while the conformation of their active sites are largely different. Analysis of all these structures indicated that they may represent different states of the enzyme in the reaction cycle. Except WT-HP structure, which is similar to that of the *Pm*ADO, the other four structures exhibit different conformations in their active sites that were not described previously.

## Results and Discussion

### Overall structure of *Se*ADO

**Figure 1:**
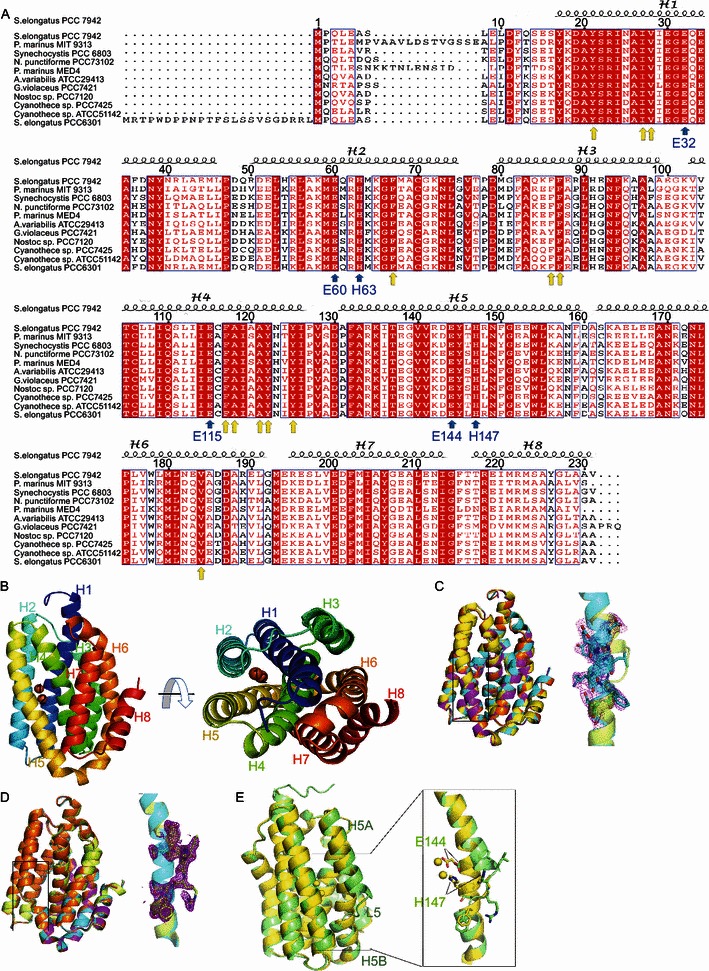
**Sequence and structure of*Se*ADO**. (A) Sequence alignment of cADOs from different species. The secondary structure elements are indicated. The residues comprising the conserved iron-binding motifs are indicated with blue arrow and labelled. The conserved residues involved in substrate channel formation are indicated with yellow arrow. (B) Overall structure of *Se*ADO (molecule A of WT1 structure), secondary structure was labelled, iron atoms are shown as spheres. (C) Structural comparison of iron-bound *Se*ADO structures. The black square marked the H5 region (left panel). The 2Fo – Fc (1.0*σ* level) electron density of this segment in WT1 structure (H5) was shown in right panel. (D) Structural comparison of iron-free *Se*ADO structures. The black square marked the L5 region (left panel). The 2Fo – Fc (1.0*σ* level) electron density of this segment in WT0 structure (L5) was shown in right panel. (E) Structural comparison of the iron-bound *Se*ADO (molecule A of Y122F structure) and the iron-free *Se*ADO (WT0 structure). The right panel shows the different conformation of H5 and L5. Residues 144–150 are shown as stick and the two iron-coordinating residues Glu144 and His147 are indicated. The WT0, Y122F, WT1, F86YF87Y and WT-HP structures are shown in lime, yellow, cyan, orange and magenta, respectively in Fig. 1C–E

*Se*ADO belongs to the di-iron protein family, with conserved sequence of two EX_28-29_EX_2_H motifs (Kurtz, 1997; Merkx et al., 2001), in which six conservative amino acids from four helices (Glu32 from helix H1, Glu115 from helix H4, Glu60 & His63 from helix H2, and Glu144 & His147 from helix H5) act as metal ligands (Fig. 1A). Interestingly, we found that not every molecule in our five *Se*ADO structures possess a fully occupied di-iron center, even though the protein was co-crystalized with 4 mmol/L ferrous ammonium sulfate. Each asymmetric unit of WT0 crystal contains one *Se*ADO molecule, and its active site appears to have lost both of its iron atoms. The other four structures all contain two molecules in an asymmetric unit, with at least one molecule containing a di-iron center (Table 1).

**Table 1:** Statistics of data processing, structure refinement and summary of different structures of *Se*ADO

	WT0	Y122F	WT1	F86YF87Y	WT-HP
PDB ID	4QUW	4RC6	4RC5	4RC7	4RC8
**Data collection**					
Space group	P4_1_2_1_2	P2_1_2_1_2_1_	P2_1_2_1_2_1_	P2_1_2_1_2_1_	P2_1_2_1_2_1_
Cell parameters (Å)	*a* = 61.7	*a* = 64.73	*a* = 61.7	*a* = 62.0	*a* = 61.7
	*b* = 61.7	*b* = 64.71	*b* = 61.8	*b* = 62.1	*b* = 61.9
	*c* = 110.5	*c* = 101.4	*c* = 124.9	*c* = 125.0	*c* = 124.8
Resolution (Å)	50.0–2.26 (2.30–2.26)	50.0–2.9 (2.95–2.9)	50.0–2.3 (2.34–2.3)	50.0–2.2 (2.28–2.2)	50.0–1.71 (1.74–1.71)
No. of unique refls	19017	9504	21848	24713	50505
*R*_merge_ (%overall/outmost shell)	3.9/36.9	6.0/50.9	13.0/49.0	17.0/71.8	7.3/48.3
*I*/*σ*(I) (overall/outmost shell)	38.8/4.8	22.5/3.4	15.1/4.0	71.5/25.2	34.7/7.9
Completeness (% overall/outmost shell)	99.8/99.8	84.9/99.3	99.8/100.0	98.5/99.1	99.8/100
**Refinement**					
*R*_factor_ (%)	20.4	25.6	20.9	20.4	18.9
*R*_free_ (%)	24.6	29.5	24.2	24.1	21.2
RMS deviations					
Bond lengths (Å)	0.011	0.011	0.012	0.009	0.007
Bond angles (º)	1.207	1.462	1.382	1.128	0.934
Mean *B* value (Å^2^)	35.3	46.8	26.2	26.7	16.3
**Structure summary**					
Iron incorporation before crystallization	No	Yes	Yes	Yes	Yes
Treatment of crystals	–	–	–	–	H_2_O_2_ soaked
No. of molecules in a.u.	1	2	2	2	2
No. of di-iron center in a.u.	0	2	1	1 + 0.5	1
Location of di-iron center	–	Molecules A and B	Molecule A	Molecules A and B	Molecule A
Ligand	L_red_ (Fatty alcohol)	–	L_red_ (Fatty alcohol)	L_red_ (Fatty alcohol)	L_ox_ (Fatty acid)
Oxo-bridge	–	No	No	No	Yes
Ligated water	–	No	No	Yes	Yes

Either with or without di-iron cluster, the overall structures of all these molecules are similar, with the exception of one specific helix conformation. In the structures of Y122F and molecule A of other threes (WT1, F86YF87Y and WT-HP), the ADO molecule adopts an all-helical folding, comprising of eight α-helices (H1–H8) that form a compact structure (Fig. 1A and 1B). Two iron atoms (Fe1 and Fe2) are surrounded by a four-helix bundle, which is composed of H1 (S16–M45), H2 (R50–L74), H4 (V103–I126) and H5 (A131–D160).

Unlike other structural elements in *Se*ADO, helix H5 is unique in representing two distinct conformations in different structures. In the structures mentioned above, H5 exists as a long helix (Fig. 1C), where two residues (Glu144 and His147) in the second EX_2_H motif are coordinated or close to iron atoms. By contrast, the helix H5 is unwound in the middle in the structures of WT0 and molecule B of other three structures (WT1, F86YF87Y and WT-HP), forming two short helices (H5A and H5B) that are connected by a loop L5 (Fig. 1D and 1E). The helix to loop transition results in a different conformation for a number of amino acids (from residue 144 to 150), thus the two iron-coordinating residues (Glu144 and His147) move away from the di-iron site (Fig. 1E). As a result, most of our *Se*ADO structures with the L5 conformation lose their metal atoms. The only exception is molecule B in the F86YF87Y structure, which contains the di-metal cluster while exhibiting the L5 conformation. However, the relatively weak electron density indicates a low occupancy of the two iron atoms in this molecule.

The fact of the partially bound iron atoms observed in our ADO structures is in agreement with previous iron content results for *Pm*ADO proteins (Das et al., 2011), as well as our ICP-OES analysis on *Se*ADO protein samples. Our analysis showed that the content of iron in ADO is relatively low when no iron was added during protein expression. Even after adding higher concentrations of iron to the culture medium, we were unable to obtain proteins with fully occupied di-iron centers (Table 2). Our results suggest that the lability of iron atoms at ADO active site is possibly related to the flexible conformation of helix H5.

**Table 2:** Iron content of wild type and mutants of *Se*ADO measured by ICP-OES assay

Protein	Expression conditions			Number of iron atom per ADO molecule	Percentage of iron content compared with wild type 3
	LB medium	TB medium	LB medium + Fe		
Wild type 1	√			0.28 ± 0.004	17.7% ± 0.25%
Wild type 2		√		0.67 ± 0.02	42.4% ± 1.27%
Wild type 3			√	1.58 ± 0.09	100% ± 5.7%
E144A			√	1.58 ± 0.06	99.9% ± 3.81%
Y122F			√	1.69 ± 0.12	107.38% ± 7.7%
F86YF87Y			√	1.30 ± 0.26	82.29% ± 16.78%
A129insI			√	1.60 ± 0.29	101.32% ± 18.17%
A129insIF			√	1.58 ± 0.03	100.25% ± 1.63%
L146S			√	1.48 ± 0.01	93.96% ± 0.76%
R148A			√	1.53 ± 0.08	96.64% ± 5.17%
N149A			√	1.35 ± 0.23	85.50% ± 14.71%

### Substrate channel in *Se*ADO

**Figure 2:**
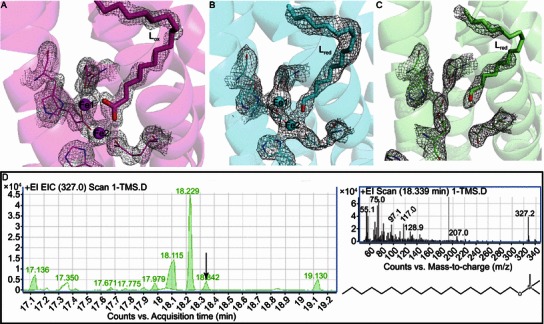
**Analysis of the ligands bound in*Se*ADO**. (A–C) The 2Fo – Fc (1.0*σ* level) electron density of the ligand, iron atoms and the conserved iron-coordinating residues in WT-HP (A), WT1 (B), and WT0 (C) structures. Iron atoms are shown in spheres, the ligands are shown in sticks and the residues are shown in lines. The WT-HP, WT1, and WT0 structures are shown in magenta, cyan, and lime, respectively. (D) GC-QqQ-MS/MS analysis and verification of the unknown ligands. The sample after extraction was trimethylsilylated for analysis on GC-QqQ-MS/MS. 1-(Trimethylsilyloxy)octadecane (CAS No.18748-98-6), the derivative of 1-hydroxyoctadecane, was identified to the peak of acquisition time 18.34 min (indicated by the black arrow)

In four of our five structures (except the Y122F structure), a continuous tube-shaped non-protein electron density, resembling a lipid molecule, was observed close to the di-iron center (Fig. 2). A similar ligand was also found in earlier *Pm*ADO structures (Khara et al., 2013; Schirmer et al., 2010). Using GC-MS analysis, Khara, et al. identified the ligand to be a mixture of long-chain fatty acids, with two components of the ligand extracts yet awaiting identification (Khara et al., 2013). In our WT-HP structure, a long-chain fatty acid molecule with a bifurcated head-group configuration fits the electron density well (Fig. 2A). However, in the WT1 and WT0 structures, the electron density is consistent with a ligand with one single-head-group configuration, which is unlikely to be a fatty acid (Fig. 2B and 2C). The differing electron densities observed in F86YF87Y structure suggest the presence of a mixture of ligands with different head groups.

To identify the precise nature of these ligands, the ligands extracted from *Se*ADO protein samples were analyzed by GC-QqQ-MS/MS. The results showed that the majority of the ligands examined were a mixture of long-chain fatty acids, and the dominant of the remaining components was identified as a C18 fatty alcohol (Fig. 2D). Following the analysis of the extracts together with the observed electron densities, we built a long-chain fatty acid (L_ox_) into the structure of WT-HP, and a long-chain fatty alcohol (L_red_) into three other structures. On the basis of these observations, we propose that the L_red_ ligand may represent the real substrate, namely fatty aldehyde, because of the similarity in their chemical structures.

**Figure 3:**
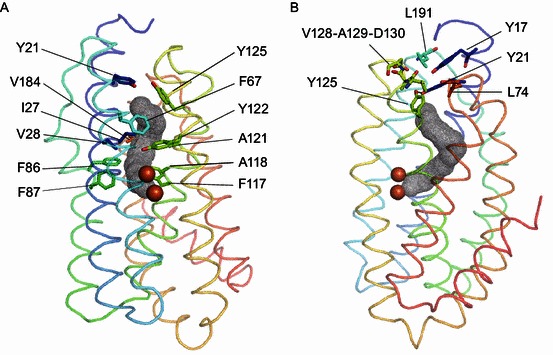
**The substrate channel of*Se*ADO**. (A) The conserved residues involved in the formation of the substrate channel are labelled. (B) The substrate channel is sealed by the hydrogen bond (shown as black dashed line) formed by two Tyr residues (Y21 and Y125) and a shell-like cover comprises of V128-D130 together with Y17, L74, and L191. The iron atoms are shown in spheres, and the substrate channel is shown as grey mesh

In our different structures, the ligand is buried inside ADO molecule at the same location, with its hydrophobic tail pointing towards the N-terminal region. A number of aromatic and hydrophobic residues, which are highly conserved among diverse species of cADOs (Fig. 1A), form a hydrophobic channel to accommodate the non-polar ligand (Fig. 3A). Notably, whilst all these residues are provided by helices 1, 2, 3, 4 and 6, helix H5 is contributing little to the formation of the hydrophobic channel. This peculiar structural feature might explain why the ligands remain in the channel even in the absence of the di-iron cluster (Fig. 2C), despite the fact that the conformation of helix H5 is vastly different among our structures. We proposed that the hydrophobic channel is the actual substrate channel of cADO.

**Figure 4:**
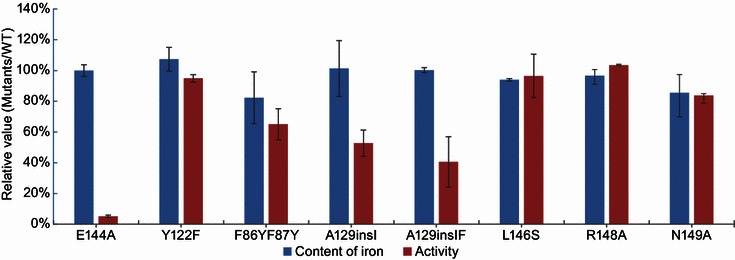
**Enzymatic activity and iron-binding capacity of cADO mutants relative to those of wild type proteins**

The analysis of our structures showed that the substrate channel is in an occluded mode. Many interactions exist between residues from several loops covering the entrance of the substrate channel in the shape of a lid (Fig. 3B). We constructed two mutants with one or two non-polar bulky residues (I or IF) inserted after A129 (A129insI and A129insIF) to lengthen the loop between H4 and H5. The enzymatic assay showed that both mutants exhibited only up to half of the activity of that observed for wild type (Fig. 4). We propose that a longer loop may lead to the steric hindrance at the entrance of substrate channel, which could result in the reduction of ADO activity. In addition, two Tyr (Y125 and Y21) form a hydrogen bond at the entrance of the substrate channel, sealing the ligand inside the protein molecule (Fig. 3). The above-mentioned structural features might be one of the reasons that cADO appears inactive when longer-chain aldehydes like C24 are used as substrate (Andre et al., 2013).

### *Se*ADO structures that represent the different states during catalytic reaction

**Figure 5:**
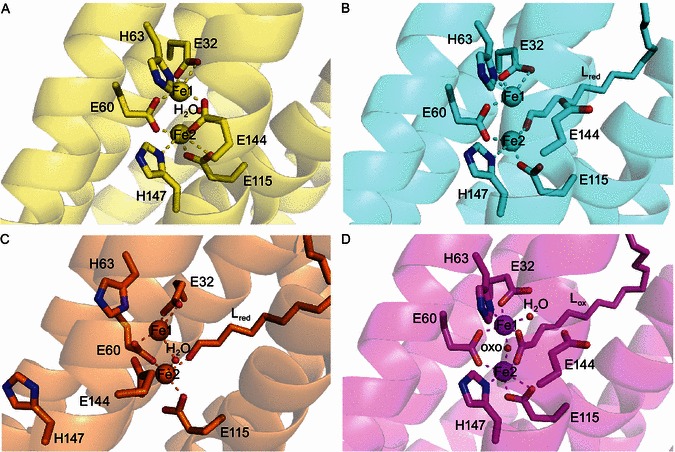
**The coordination of di-iron center in the structures of Y122F (A), WT1 (B), F86YF87Y (C), and WT-HP (D)**. Iron atoms are shown in spheres, the coordinating residues and ligand are shown in sticks. The structures of Y122F, WT1, F86YF87Y, and WT-HP are shown in yellow, cyan, orange, and magenta, respectively

In order to test the effects of residues surrounding the di-iron center and substrate channel on cADO’s catalytic activity, we constructed several mutant forms by site-directed mutagenesis, and then determined the iron content and enzymatic activity of these mutants relative to the wild type (Fig. 4 and Table 2). In addition, the crystal structures of specific mutants were solved. Intriguingly, two mutant structures (Y122F and F86YF87Y) and three wild type structures exhibit different conformations at their active site (Fig. 5) which may represent different states of the enzyme in the reaction cycle. Based on the character of the ligand molecules and the coordination mode of the iron atoms, we assign our five structures in a different states in ADO catalytic reaction below.

#### The substrate-free structure of Y122F represents the initial state of the reaction

In the Y122F structure, very little electron density was observed in the substrate channel, which is in contrast to all other four structures. However, there is still a little electron density remaining in the vicinity of the di-iron site, which we assigned to a water molecule forming a hydrogen-bond interactions with the carboxylic oxygen atoms of Glu144 and Glu115. This water molecule is not coordinated to either iron ions, with a distance of 3.28 Å and 3.15 Å, respectively. Apart from harboring the vacant substrate channel, the Y122F mutant shows comparable enzymatic activity with wild type. This result is consistent with that of the equivalent Y135F mutant in *Pm*ADO (Schirmer et al., 2010). In the Y122F structure, the di-iron site is surrounded by the four-helix bundle, each iron ion is penta-coordinated by two bridging carboxylates ligands (Glu60 and Glu144), one bidentate carboxylates and one histidine ligand (Glu32 and His63 for Fe1, Glu115 and His147 for Fe2) (Fig. 5A and Table 3). The coordination geometry of the two iron atoms is similar to that of other di-iron proteins in their rest state of reduced forms (Du Bois et al., 2000; Eriksson et al., 1998; Hogbom et al., 2002; Lindqvist et al., 1996; Logan et al., 1998; Logan et al., 1996; Strand et al., 2004; Yang et al., 2000) which is consistent with the initial state of cADO in the reaction cycle. We assumed that this structure with a vacant substrate channel represents the initial and substrate-free status during the reaction cycle.

**Table 3:** Coordinating ligands and distances of two iron atoms in *Se*ADO structures

	Y122F (A)	WT1 (A)	F86YF87Y (B)	WT-HP (A)*
*Fe1*				
E32 (OE1)	2.07	2	2.25	2.05
E32 (OE2)	2.29	2.44	2.22	
H63	2.38	2.35		2.25
E60 (OE1)	1.91	1.99	2.17	2.0
E144 (OE2)	2.10	–		–
Water2 (only in F86YF87Y)		–	2.46	
Water1		–		1.94
OXO		–		1.81
LIGAND		–		2.08
*Fe2*				
E115 (OE1)	2.33			2.38
E115 (OE2)	2.43	2.05	2.11	2.14
H147	2.13	2.3		2.25
E60 (OE2)	2.13	2.06	1.73	2.04
E144 (OE1)	1.91	–		–
Water2 (only in F86YF87Y)			2.2	
OXO		–		1.92
Ligand		2.29	2.16	2.31

* Molecule A of Y122F, WT1 and WT-HP structures, and molecule B of F86YF87Y structure were used to measure the distances

#### The WT1 structure represents the state of ADO bound with substrate

In the WT1 structure, a L_red_ ligand, which was proposed to mimic the substrate aldehyde, was modeled in the substrate channel according to the electron density (Fig. 2B). The ligand approaches the di-iron cluster from the opposite side of His147, with its head-group binding directly to the Fe2 atom. This coordination mode suggests that Fe2 is the preferred iron for substrate binding. The water molecule, located near the di-iron site in the Y122F structure, is displaced by the head group of the L_red_ ligand. The residue Glu144, which is located in helix H5, changes its rotamer and is not coordinated with either iron atoms. In addition, E115 alters its mode of Fe2 coordination, from bidentate to monodentate, while other four iron-coordinating residues stay unchanged, resulting in a distorted tetrahedral coordination of both Fe1 and Fe2 (Fig. 5B and Table 3). The structure of WT1 is likely to represent the state where the enzyme is bound with substrate. The structural comparison between WT1 and Y122F implied that the swing of Glu144 might be induced by ligand binding, thus enabled us to visualize the conformational change coupled with substrate binding in cADO active site for the first time.

#### The F86YF87Y structure represents the state of ADO where the oxygen path is formed

**Figure 6:**
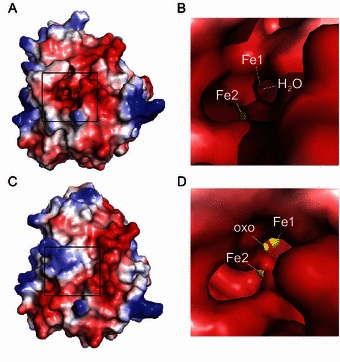
**Electrostatic surface interpretation of*Se*ADO structures**. (A) Surface representation of F86YF87Y structure showed that a hole was formed in its surface due to the conformational change of helix H5. (B) The partial enlarged detail of surface interpretation of F86YF87Y structure, two iron atoms (shown as yellow spheres) and the water molecule (shown as red sphere) in the active center can be observed from the hole. (C) Surface representation of WT-HP structure. (D) The partial enlarged detail of surface representation of F86YF87Y structure with the di-iron cluster of WT-HP structure superimposed. The oxo bridge and the two iron atoms (shown as yellow spheres), which are hidden inside the ADO molecule in WT-HP structure (C), are accessible in F86YF87Y structure (D)

In the molecule B of F86YF87Y structure, a segment (from residue 144–150) in helix H5 changes its conformation from helix to loop, which might be induced by the swing of Glu144 observed in WT1 structure. As a consequence, His147 moves away from the active site with a 9Å distance to Fe2. In addition, H63 is located more distantly from the active site, and the ligand bond between Fe1 atom and H63 is broken. The iron ions at the active site are bridged by the residue Glu60 together with a water molecule. However, the water molecule is in a different location from the one observed in the Y122F structure. The two iron atoms are maintaining their tetrahedral coordinations (Fig. 5C and Table 3). The relatively low coordination number in this structure indicates that the active site is in a reduced state, and should be highly sensitive to dioxygen (Nordlund and Eklund, 1995). In addition, a hole is formed at the protein surface as a result of helix H5 distortion, which exposes the di-iron cluster and the bridging water molecule to solvent (Fig. 6A and 6B). We assumed that the hole formed in the F86YF87Y structure serves as the access channel for the co-substrate dioxygen. Recent research revealed that the substrate-free ADO is relatively unreactive towards O_2_, and its O_2_ reactivity is triggered by substrate binding, which is referred to as a “substrate triggering” mechanism (Pandelia et al., 2013). Based on our structural analysis, we proposed that the substrate triggering is due to the conformational changes of Glu144 and helix H5 induced by substrate binding to create a path for O_2_. Therefore, we presumed that this structure represents the transient state at which ADO is awaiting the arrival of the co-substrate dioxygen.

#### The WT-HP structure represents an intermediate state of ADO where oxygen reacts with the substrate

To explore the effects of H_2_O_2_ on ADO activity, a crystal of wild type *Se*ADO was soaked in H_2_O_2_, and its structure (WT-HP) was solved. No electron density was visible for the H_2_O_2_, probably due to its unstable nature. A L_ox_ ligand closely resembling a fatty acid molecule was built according to the electron density observed within this structure (Fig. 2A), which is nearly identical to the previously solved structure of *Pm*ADO (PDB code 2OC5). In the WT-HP structure, the helix conformation of H5 was restored, and the two coordinating residues His63 and His147 approach the active site and ligate the Fe1 and Fe2 atoms, respectively. The water molecule that bridges the two iron atoms in the F86YF87Y structure moves towards Fe1 and is not coordinated with Fe2 in the WT-HP structure. The two iron ions are bridged by one carboxylate ligand E60, the L_ox_ ligand and an oxo group. Both iron atoms show saturated coordination with an octahedral geometry (Fig. 5D and Table 3). The higher coordination numbers of di-iron center, together with the fact that the crystal was obtained after being treated with strong oxidizer H_2_O_2_, lead to the speculation that the WT-HP structure represents an oxidized form of ADO. Furthermore, the coordination mode of two iron atoms in WT-HP structure shows a similar pattern to that observed for the R2 protein of ribonucleotide reductase (RNR-R2), in which the oxo bridge and the coordinated water exist at the di-iron site in the oxidized structure, while both are absent in the reduced structure (Logan et al., 1996). The superimposition of the F86YF87Y and WT-HP structures revealed that the hole at the protein surface, which is caused by the distortion of helix H5, exposes the di-iron sites and the oxo bridge (Fig. 6C and 6D). This observation supports our hypothesis that the identified hole serves as dioxygen channel. We proposed that the L_ox_ ligand is likely to be the analog of the possible intermediate product hemiacetal, thus the WT-HP structure may represent the intermediate state in reaction of cADO, following the entering of dioxygen and its reaction with the substrate.

#### The WT0 structure represents the inactive state of ADO

The WT0 structure is characterized by losing the di-iron cluster and by exhibiting a distorted conformation of helix H5. This structure is likely to represent the inactive state of *Se*ADO, as it has lost its cofactor iron. We assume that if the loop conformation of helix H5, as revealed in the F86YF87Y structure, was not restored into a helical structure at the appropriate time, the solvent-accessible di-iron center may become unstable. Whereas the instability of the di-iron cluster further promotes the loop conformation, and thus results in a complete loss of the di-iron cluster, as shown in the WT0 structure (Fig. 1D and 1E). The structural flexibility of *Se*ADO provides an explanation for its low iron occupancy, and may be in part responsible for the low enzymatic activity observed for cADO. To the best of our knowledge, the observation that the conformational change of one specific helix towards the loop correlates with the loss of the di-iron cluster is the first case described for this superfamily.

### The proposed process of the catalytic reaction by cADO

Among all the iron-coordinating residues, Glu144 remarkably alters its conformation among structures. A similar case was observed in other members of the di-iron protein family, such as RNR-R2 and the hydroxylase component of soluble methane monooxygenase. Both possess an essential iron-coordinating glutamic acid, which exhibits distinct conformations during their respective reactions (Kolberg et al., 2004; Sazinsky and Lippard, 2006; Whittington and Lippard, 2001). Apart from the structural information, our biochemical analysis showed that the E144A mutant has only 5% activity remaining, with similar iron content as measured for the wild type (Fig. 4 and Table 2). We therefore suggest that Glu144 plays an important role in catalysis.

**Figure 7:**
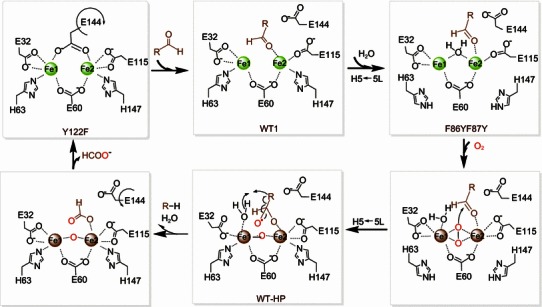
**Proposed catalytic process of cADO based on the structures presented in this work**

Based on our structural and biochemical analysis, together with the previous report on ADO (Li et al., 2012; Li et al., 2011; Pandelia et al., 2013; Paul et al., 2013; Warui et al., 2011), we proposed a potential process of catalytic reaction for cADO (Fig. 7). Initially, the substrate ligates to the Fe2 and induces the swing of Glu144 away from the di-iron site and the conformational change of helix H5. The swing of Glu144 is essential for both inducing the distortion of H5 that facilitates the formation of the O_2_-entering path, and makes room for subsequent dioxygen coordination. Following the entering of O_2_ into the active site, the helical conformation of H5 is restored. Meanwhile, one oxygen atom forms the oxo bridge that ligates both iron atoms, and the other oxygen atom attacks the substrate aldehyde to form the intermediate product of hemiacetal. Then the scission of its C1–C2 bond occurs, with the bond likely to be under the attack of the Fe1-coordinating water, and this attack results in the release of the product alkane. Finally, Glu144 shifts back to the active site, bridging two iron atoms. The oxo-bridge is broken, and the product formate is released, together with one water molecule.

In summary, we determined five crystal structures of *Se*ADO and revealed novel structural features around their active site. Snapshots of these consecutive states allow us to visualize the morphing of the active site during the reaction. Analysis of our structural and biochemical data highlights a number of important structural features that can influence the catalytic process and activity of cADO, including the conformational switch of the central part of helix H5, and the flexibility of residue Glu144. Together, these results provide new structural insights into the catalytic mechanism and allow us to propose a possible catalytic process of cADO, thus provides crucial information required for developing new strategies to improve its enzymatic activity, with the ultimate goal of producing fuel-grade alk(a/e)nes in a renewable and sustainable manner.

## Materials and Methods

### Protein expression and purification

The codon-optimized gene encoding cADO from *Synechococcus elongates* PCC7942 was synthetized and cloned into the expression vector pET-28a(+) (Novagen). The constructs for mutant of *Se*ADO were generated using the QuikChange site-directed mutagenesis kit (Stratagene). The constructs were confirmed by DNA sequencing and transformed into *E. coli* BL21(DE3). Protein expression was induced by adding isopropyl β-D-thiogalactoside (IPTG) to a final concentration of 1 mmol/L. After being shaken at 37°C for approximately additional 4 h, cultures were harvested by centrifugation at 6000 ×*g* at 4°C for 15 min. To obtain the iron-bound cADO proteins, 2 mmol/L (NH_4_)_2_SO_4_FeSO_4_·6H_2_O was added in medium.

The cell pellet was homogenized in buffer containing 20 mmol/L Tris-HCl pH 7.8 and 300 mmol/L NaCl (buffer A), and sonicated. Cell debris was removed by centrifugation at 40,000 ×*g* for 30 min. The supernatant was collected and loaded onto Ni-IDA resin (Chelating sepharose FF, GE Healthcare) and rinsed with buffer A 20 mmol/L imidazole. The protein was eluted from the affinity resin with buffer A containing 250 mmol/L imidazole. The eluted fraction was concentrated and further purified by gel filtration on Superdex 75 (GE Healthcare) with elution buffer 50 mmol/L Hepes pH 7.2, 150 mmol/L NaCl. The purified protein was concentrated to 10 mg/mL for crystallization.

Ferredoxin reductase and ferredoxin derived from *Synechococcus elongates* PCC7942 were constructed and purified as previously described (Zhang et al., 2013).

### Crystallization and X-ray data collection

Crystallization trials were carried out at 18°C by mixing equal volume of protein and reservoir solution using the sitting-drop vapor diffusion method. Crystals of WT0 structure was harvested in condition of reservoir solution of 0.2 mol/L Magnesium chloride hexahydrate, 0.1 mol/L Tris hydrochloride pH 8.5, 30% (*w*/*v*) PEG 4,000. To obtain the iron-bound crystals, 4 mmol/L ferrous ammonium sulfate was added to *Se*ADO protein solutions right before crystallization. The crystals of WT1 structure were harvested in reservoir solution containing 0.2 mol/L L-proline, 0.1 mol/L Hepes pH 7.1, 25% (*w*/*v*) PEG1500. The crystals of Y122F and F86YF87Y were grown in the same solution with 0.02 mol/L adenosine-5’-triphosphate disodium salt hydrate added. The crystal of WT1 was soaked in 10 mmol/L H_2_O_2_ for 30 min before being flash frozen to obtain the crystal of WT-HP.

Crystals were flash-cooled in a nitrogen-gas stream at 100 K for data collection. Diffraction data of Y122F and F86YF87Y were collected on beamline BL17U at Shanghai Synchrotron Radiation Facility. Diffraction data of WT0 and WT1 were collected utilizing Rigaku RAXIS IV image plate detector at Institute of Biophysics (Chinese Academy of Sciences). Diffraction data of WT-HP were collected on BL17A at Photo Factory, Japan. Diffraction data were processed and scaled with HKL-2000 package (Otwinowski and Minor, 1997).

### Structure determination and refinement

The WT0 model, which subsequently make a searching model for molecular replacement of other four structures, was solved by molecular replacement with Phaser_MR (Mccoy et al., 2007) using the *Pm*ADO structure (PDB code 2OC5) as a searching model. The model were rebuilded by AutoBuild in PHENIX package (Adams et al., 2010), following subjected to refinement by Phenix.refine (Adams et al., 2010) and COOT (Emsley et al., 2010). Figures of the structures were prepared by Pymol (DeLano Scientific, LLC). A summary of data collection and structure refinement statistics is given in Table 1.

### Metal content determination and enzyme activity assay

The metal contents of the wild-type enzyme and mutants were determined by inductively coupled plasma optical emission spectrometer (ICP-OES), PerkinElmer 5300DV.

N-heptanal was selected as the substrate to measure the activity of cADO proteins. Assays were performed in 1.5 mL gastight vials with a total volume of 500 μL, and reactions were conducted in 100 mmol/L HEPES buffer pH 7.2 containing 100 mmol/L KCl and 10% glycerol, with 15 μmol/L protein samples, 2 mmol/L of n-heptanal in final 4% DMSO, 30 μg/mL ferredoxin, 0.04 U/mL ferredoxin reductase, 800 μmol/L NADPH, 60 μmol/L ferrous ammonium sulphate. After being enrolled of all the components, reactions were shaken at 220 rpm at 37°C for 30 min. To determine the amount of *n*-hexane produced, a sample of the headspace was collected using a gastight sample lock Hamilton syringe and analysed by Shimadzu GC-2010 with DB-5 column. The amount of *n*-hexane produced was quantified by a standard curve of known concentrations of *n*-hexane.

For GC analysis the flow rate of the nitrogen carrier gas was 1.1 mL/min and the inlet temperature was maintained at 220°C. Injections were made in split mode with a split ratio of 2:1 and a total flow of 2 mL/min. The oven temperature was held at 40°C for 3 min and then increased to 120°C at 10°C/min, and finally maintained at 120°C for 2 min. The FID detector was at 250°C with a continuous flow of H2 at 40 mL/min and air at 400 mL/min. Chromatographic data were analyzed using the associated software.

### Determination of ligand(s) using GC-QqQ-MS/MS.

2 g of purified *Se*ADO protein was acidified using 1 mol/L HCl to pH 3.0 and extracted with ethyl acetate. The organic layer was collected and dried by passing through MgSO_4_. The solvent was evaporated by rotary evaporator and nitrogen (N-EVAP) to 100 μL. The sample was trimethylsilylated for analysis using BSTFA + 1% TMCS (Sigma). All spectra were recorded on an Agilent 7890A GC system connected to an Agilent 7000B triple quadrupole MSD with electron impact ionization mode. A 1-μL portion of the derivatized extract was injected in splitless mode onto the column. The column used was a DB-5ms (30 m × 250 μm × 0.25 μm film thickness, Agilent J&W ScientiWc, USA) fused silica capillary column. Injector temperature was 280°C and the oven program was as follows: oven temperature was held at 60°C for 2 min and then increased to 240°C at 10°C/min and then increased to 300°C at 20°C/min finally maintained at 300°C for 5 min. Helium was used as the carrier gas for GC at a flow rate of 1.0 mL/min, and the inlet temperature was maintained at 280°C with splitless. Chromatographic data were acquired and processed using MassHunter Workstation Quantitative Software.

## Abbreviations

AAR: acyl-ACP reductase
ADO: aldehyde-deformylating oxygenase
cADO: ADO from cyanobacteria
Fd/FR/N: ferredoxin, ferredoxin reductase, and NADPH
ICP-OES: inductively coupled plasma optical emission spectrometer
IPTG: isopropyl β-d-thiogalactoside
P/N: phenazine methosulfate and NADH
Pm: *Prochlorococcus marinus* MIT9313
RNR-R2: the R2 protein of ribonucleotide reductase
Se: *Synechococcus elongates* PCC7942
